# Treatment Satisfaction with Subcutaneous Immunoglobulin Replacement Therapy in Patients with Primary Immunodeficiency: a Pooled Analysis of Six Hizentra® Studies

**DOI:** 10.1007/s10875-018-0562-3

**Published:** 2018-11-21

**Authors:** Rajiv Mallick, Stephen Jolles, Hirokazu Kanegane, Dominique Agbor-Tarh, Mikhail Rojavin

**Affiliations:** 1 CSL Behring LLC, King of Prussia, PA USA; 2 University Hospital of Wales, Cardiff, UK; 3 Department of Child Health and Development, Graduate School of Medical and Dental Sciences, Tokyo Medical and Dental University, Tokyo, Japan; 4 Frontier Science, Kincraig, Scotland UK

**Keywords:** Quality of life, subcutaneous immunoglobulin replacement therapy, immunodeficiency

## Abstract

**Purpose:**

Primary immunodeficiency diseases (PIDDs) are a heterogenous group of disorders characterized by intrinsic impairment in the immune system. Most patients with PIDD require life-long immunoglobulin G replacement therapy, which has been shown to reduce the rate of infections and, related hospitalizations and reduce health-related quality of life (HRQOL). Here, treatment satisfaction and HRQOL in patients with PIDD was evaluated upon switching from intravenous (IVIG) or subcutaneous immunoglobulins (SCIGs) to 20% SCIG (Hizentra®), and during long-term steady-state Hizentra® treatment.

**Methods:**

Analyses were based on two pivotal (switch) and four extension/follow-up (maintenance) Phase III studies of Hizentra® conducted in Europe (EU), Japan (JP), and the United States (US). Two validated questionnaires were used: Life Quality Index (LQI) for assessment of IgG-specific perceptions of HRQOL and Short Form 36 version 2 (SF-36v2).

**Results:**

In the EU and JP switch studies, there was significant and meaningful improvement from Screening in LQI domain scores at all time points, largely driven by patients switching from IVIG to SCIG. In the EU switch study, there were also significant increases in mean SF-36v2 domain scores for Physical Function and General Health from Screening to Week 12. These improvements were observed also at Week 24. Overall, LQI and SF-36v2 domain scores were generally sustained in the maintenance studies.

**Conclusions:**

These results showed that switching patients from IVIG to SCIG improves patient self-reported health status and IgG-specific HRQOL perception. The maintenance studies generally showed no deterioration of this improved health status over a long follow-up period.

**Electronic supplementary material:**

The online version of this article (10.1007/s10875-018-0562-3) contains supplementary material, which is available to authorized users.

## Introduction

Primary immunodeficiency diseases (PIDDs) consist of a heterogenous group of disorders in which there is an intrinsic impairment in the body’s immune system [1, 2]. While up to 300 types of PIDD are recognized, common variable immunodeficiency and X-linked agammaglobulinemia are two of the most common [1, 2]. The true prevalence of PIDD is not well established, but estimates suggest that PIDD affects 1 in 2000 children, 1 in 1200 individuals of any age, and 1 in 600 households in the United States (equivalent to 150,000–360,000 patients) [3].

PIDD imposes a significant disease burden on patients, including limitations in work, play, and normal physical activity [4]. In comparison with healthy children and adults, patients with PIDD experience measurably lower general health with higher hospitalization rates and increased physical, school, and social activity limitation [5–7]. Further, patients with PIDD fare even worse than those with other chronic conditions with respect to general health perceptions [8, 9].

Fortunately, effective treatment of PIDD is available and associated with patient benefit. Most patients with PIDD require life-long immunoglobulin G (IgG) replacement therapy, which has been directly shown in longitudinal studies to decrease not only the rate of life-threatening bacterial infections and related hospitalizations [10, 11], but also improve health-related quality of life (HRQOL) [12].

Since IgG therapy can be administered either intravenously (IVIG) or subcutaneously (SCIG) [13–16] and constitutes life-long treatment, the choice of modality of administration can have major implications for patient well-being over their lifetime [17]. Evidence suggests that the efficacy of IgG therapy is similar whether it is administered as SCIG or IVIG [18, 19], yet compared to IVIG, SCIG offers the benefits of more stable serum IgG levels, increased patient flexibility due to self-administration and flexible dosing schedules, and a lower rate of systemic adverse events (AEs) [20, 21]. Furthermore, pharmacokinetic modeling has previously shown that doses can be administered at varying intervals (daily to biweekly) with little impact on serum IgG levels [22, 23].

SCIG, which has been gaining significant favor as an administration route [22, 23], is almost always administered in a home setting. It has the potential to improve several important aspects of HRQOL in patients with PIDD, including fewer limitations with regard to work or other daily activities and reduced impact of treatment on everyday life [21].

There are a variety of SCIG formulations available, including those with IgG concentrations of 10%, 16%, or 20%, and a recombinant human hyaluronidase-facilitated SCIg (fSCIg) [18, 24, 25]. Hizentra® (IgPro20, CSL Behring, Bern, Switzerland) was the first 20% liquid IgG product for subcutaneous administration [26]. The high IgG concentration in Hizentra® allows for a small infusion volume and a short infusion time, thereby further increasing flexibility of dosing and potentially patient HRQOL [21, 27, 28].

Herein, we report results from a pooled analysis of HRQOL results from two pivotal Phase 3 studies and four follow-up/extension studies in patients with PIDD from Europe (EU), the United States (US), and Japan (JP), upon switching from IVIG or another SCIG to Hizentra® and during long-term maintenance Hizentra® treatment.

## Methods

### Patients and Study Designs

This pooled analysis included HRQOL data from six prospective, open-label, multicenter, single-arm, Phase 3 studies of efficacy and safety of Hizentra® in patients with PIDD: EU pivotal (NCT00542997) [29] and extension (NCT00751621) [15] studies; JP pivotal (NCT01199705) [30], follow-up (NCT01458171), and extension (NCT01461018) studies; US extension (NCT00719680) [15] study. HRQOL was not assessed in the US pivotal study (NCT00419341) [28]. The clinical study protocols, informed consent forms, and any other appropriate study-related documents were reviewed and approved by an Independent Ethics Committee (IEC)/Institutional Review Board (IRB) for the individual studies.

The pivotal studies were designed to evaluate a switch to Hizentra® from IVIG or other SCIG (hereafter referred to as “switch studies”), while the follow-up/extension studies were designed to evaluate long-term continuation of Hizentra® therapy (hereafter referred to as “maintenance studies”). Patients in the EU switch study could switch from IVIG or another SCIG to Hizentra®, whereas all patients in the US and JP switch studies switched from IVIG to Hizentra®.

The study design and methods of all seven studies have been previously reported [15, 26, 28, 30]. Inclusion criteria for the switch studies included confirmed PIDD previously treated with IVIG at 3–4 weekly intervals (JP switch study: for at least three doses; US switch study: for at least 3 months), the EU switch study included patients on IVIG or SCIG at regular weekly intervals, both for at least 6 months. Patients were aged 2–75 years in all switch studies (JP: aged ≤ 75 years; EU: aged 2–65 years [16–65 in UK]; US: aged 2–75 years).

Major exclusion criteria for the switch studies included newly-diagnosed PIDD (i.e., not having received previous IgG replacement therapy); serious bacterial infection (SBI) at the time of screening or first infusion; malignancies of lymphoid cells such as lymphocytic leukemia, non-Hodgkin’s lymphoma, and immunodeficiency with thymoma; a positive result at screening for any of the following viral markers: human immunodeficiency virus, hepatitis C virus, or hepatitis B virus.

For the maintenance studies, inclusion criteria included confirmed PIDD and participation in the preceding switch study. Exclusion criteria for these studies included ongoing SBIs at the time of first infusion, hypoalbuminemia, protein-losing enteropathies, and any proteinuria.

Patients enrolled in the studies received weekly subcutaneous infusions of Hizentra®. Several infusions were performed under supervision at the study site, the rest were administered at home by the patient or patient’s caregiver.

### Patient-Reported Outcomes

Two validated questionnaires were used: the Life Quality Index (LQI) for assessment of IgG-specific perceptions of HRQOL and the Short Form 36 version 2 (SF-36v2) [21] for assessment of general health status (patient functioning and wellbeing).

The LQI is an instrument specifically designed to evaluate perceptions of HRQOL, among patients receiving IgG treatment [31]. The LQI questionnaire consists of 15 treatment-specific items developed to examine the impact of IgG treatment on patients’ convenience, comfort, and independence, as well as assessing the impact of treatment schedule flexibility, pleasantness of treatment setting, disruption of daily activities, treatment-related time involvement, and cost burden. The LQI is summarized into four domains: Treatment Interference, Therapy-related Problems, Therapy Setting, and Treatment Costs. The LQI domains are scored from 0 to 100, with higher scores associated with better IgG treatment-specific HRQOL. LQI was evaluated at the following time points (Fig. S1): Screening, Week 12, Week 24, and Week 40 in the EU switch study; Month 6, 12, 18, 24, 30, 36, 42, or Study End (defined as the last available post-Screening observation for each patient) in the EU maintenance study; Screening, Week 12, and Week 24 in the JP switch and JP maintenance studies; and Week 1 and Week 60 in the US maintenance study.

The SF-36v2 is a generic tool that assesses health status using 36 items across 8 domains: Physical Functioning, Role Physical, Bodily Pain, General Health, Vitality, Social Functioning, Role-Emotional, and Mental Health, as well as an item on Reported Health Transition. Following standard scoring of the SF-36v2, raw scores on each domain were transformed to a 0 to 100 scale (higher scores representing better health status), using the following formula:

$$ \left(\frac{\mathrm{actual}\;\mathrm{raw}\;\mathrm{score}-\mathrm{lowest}\ \mathrm{possible}\ \mathrm{raw}\;\mathrm{score}}{\mathrm{highest}\ \mathrm{possible}\ \mathrm{raw}\ \mathrm{score}-\mathrm{lowest}\ \mathrm{possible}\ \mathrm{raw}\ \mathrm{score}}\right)\times 100 $$

Normalizing the results to known US or UK populations (i.e., using T-scores) was not performed, as the studies were not specific to these countries. The SF-36v2 questionnaire was administered at the same time points as the LQI, with the exception of the US maintenance study (at Week 0, 24, 48, 72, and 96; Fig. S1).

### Statistical Analysis

All analyses were conducted using SAS version 9.4 (SAS Institute Inc., Cary, NC, US). Comparisons across time points were made by fitting a linear mixed effect model on repeated measurements, with compound symmetry as the covariance structure and visit as the only explanatory variable.

Changes from Screening were estimated using least squares (LS) means derived from this model and are, therefore, not identical with simple numerical differences between means. Significance of change scores on the various domains of the LQI and the SF-36v2 were not adjusted for multiplicity. No substantive changes in the model results were seen with other correlation structures tested.

In addition to statistical significance, the clinical meaningfulness of each of the changes in the switch studies was evaluated based on within-group Cohen effect sizes [32]. Effect sizes in the range 0.2–0.5 were deemed at least minimally meaningful, 0.5–0.8 moderately meaningful, and those above 0.8 highly meaningful [32].

The analysis populations for both LQI and SF-36v2 assessments consisted of all patients in the HRQOL set (all enrolled patients with screening and ≥ 1 follow-up HRQOL assessments; EU switch and maintenance studies, US maintenance study) or the full analysis set (FAS; all patients treated with Hizentra® during the efficacy period; JP switch and maintenance studies). Questions from LQI and SF-36v2 were analyzed individually and by domain.

## Results

### Patient Disposition

Out of a total of 125 treated patients and 108 patients with efficacy data, a total of 92 unique patients (37 females and 55 males) receiving a total of 12,453 infusions were included in the HRQOL analysis sets (those with completed HRQOL assessments). The age range was 3–69 years. Demographic and Screening clinical characteristics of patients from each study are summarized in Table 1**.**

**Table 1:** Patient characteristics at Screening (populations used for HRQOL evaluation)

	EU switch	EU maintenance	JP switch	JP maintenance^a^	US maintenance
Total number of patients	51	40	24	23	17
Gender, *n* (%)					
Female	16 (31.4)	12 (30)	9 (37.4)	9 (39.1)	12 (70.6)
Male	35 (68.6)	28 (70)	15 (62.5)	14 (60.9)	5 (29.4)
Age (years)					
Mean (SD)	22.6 (15.86)	21.6 (15.31)	20.5 (13.5)	20.8 (13.68)	45.1 (16.03)
Median (range)	18 (3, 60)	16.0 (4, 52)	17.5 (3, 58)	17.0 (4, 58)	44 (11, 69)
Body mass index (kg/m^2^)					
Mean (SD)	20.64 (4.66)	20.54 (4.67)	18.8 (3.74)	18.9 (3.19)	27.7 (6.24)
Median (range)	20.2 (12.3, 31.8)	20.55 (13.9, 31.4)	18.2 (15, 33)	18.4 (15, 30)	28 (17.6, 42.7)
Primary disease, *n* (%)					
CVID	30 (58.8)	23 (57.5)	10 (42.0)	10 (43.5)	17 (100)
XLA	20 (39.2)	16 (40.0)	12 (50.0)	11 (47.8)	–
ARAG	1 (2.0)	1 (2.5)	1 (4.2)	1 (4.3)	–
LQI domain score at Screening, mean (SD)					
Treatment Interference	69.25 ± 21.77	83.76 (16.00)	52.78 (22.22)	73.91 (16.30)	83.18 ± 14.15
Therapy-Related Problems	72.64 ± 20.16	80.56 (14.97)	56.50 (21.35)	63.59 (17.37)	77.78 ± 16.17
Therapy Setting	72.96 ± 24.73	89.60 (15.46)	56.89 (22.24)	78.99 (19.67)	87.96 ± 13.10
Treatment Costs	58.33 ± 30.53	66.67 (22.21)	46.33 (27.12)	71.74 (18.93)	84.26 ± 18.05
SF-36v2 domain score at Screening, mean (SD)					
Physical Functioning	86.97 ± 17.23	92.95 ± 7.51	–	–	78.24 ± 23.91
Role Physical	78.60 ± 22.75	84.66 ± 22.55	–	–	81.99 ± 21.30
Bodily Pain	74.97 ± 23.04	82.84 ± 20.93	–	–	73.53 ± 20.81
General Health	42.82 ± 17.37	50.00 ± 19.52	–	–	50.00 ± 20.77
Vitality	58.90 ± 21.37	65.06 ± 13.59	–	–	56.25 ± 16.68
Social Functioning	84.85 ± 18.42	89.20 ± 12.96	–	–	78.68 ± 22.86
Role Emotional	84.60 ± 18.30	91.29 ± 17.91	–	–	85.29 ± 24.57
Mental Health	76.21 ± 11.39	80.00 ± 11.13	–	–	70.29 ± 15.46

*ARAG* autosomal recessive agammaglobulinemia, *AT* all-treated, *CVID* common variable immune deficiency, *FAS* full analysis set, *HRQOL* health-related quality of life, *ITT* intention-to-treat, *LQI* Life Quality Index, *n* number of patients, *n.a.* data not available, *SD* standard deviation, *SF-36v2* Short Form 36 version 2, *XLA* X-linked agammaglobulinemia

^a^Study includes data from two studies: JP follow-up (NCT01458171) and extension (NCT01461018) studies

### Switch Studies

#### LQI Scores from Individual Switch Studies

**Fig. 1:**
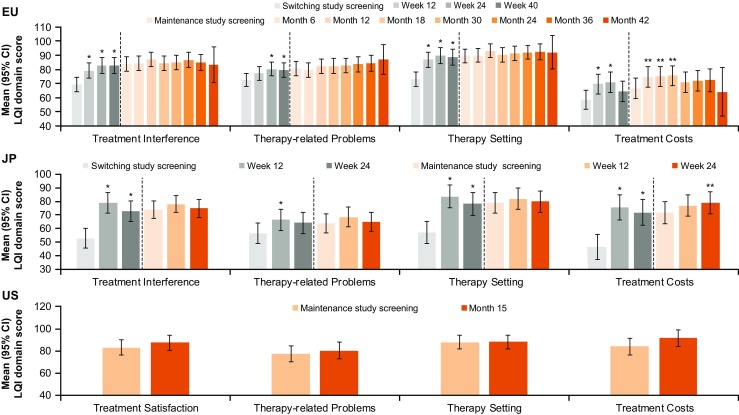
LQI domain scores in EU, JP, and US studies. Data are expressed as mean (95% CI). *p < 0.05 vs switch study Screening; **p < 0.05 vs maintenance study Screening. CI confidence interval, EU European, JP Japanese, LQI Life Quality Index, US United States

In the EU and JP switch studies, there was a significant increase (improvement) from Screening in LQI domain scores at all time points (Table 2; Fig. 1a, b). In both studies, there was a significant improvement from Screening in the mean domain scores for Treatment Interference, Therapy Setting, and Treatment Costs at Week 12 and Week 24, and for Therapy-Related Problems at Week 12 (Table 2). Changes in the domains of Treatment Interference and Therapy Setting were mostly moderately meaningful at all time points, while those in Therapy-Related Problems and Treatment Costs were minimally meaningful. In the JP switch study, changes in all domains except Therapy-Related Problems (minimally-to-moderately meaningful changes) were highly meaningful (Table 2).

**Table 2:** LQI domain scores in EU and JP switch studies

LQI domain by visit	EU switch				JP switch			
	*N*	Change from Screening, mean (SD)	*p* values of change^a^	Effect size (meaningfulness of change)	*N*	Change from Screening, mean (SD)	*p* values of change^a^	Effect size (meaningfulness of change)
Treatment Interference (0–100)								
Week 12	47	9.91 (20.49)	*0.0012*	0.48 (minimally)	24	26.18 (19.42)	*< 0.0001*	1.35 (highly)
Week 24	44	13.51 (20.27)	*< 0.0001*	0.66 (moderately)	24	20.04 (19.42)	*< 0.0001*	1.03 (highly)
Therapy-Related Problems (0–100)								
Week 12	47	4.56 (16.89)	0.0662	0.27 (minimally)	24	9.86 (19.91)	*0.0193*	0.50 (moderately)
Week 24	44	7.50 (16.72)	*0.0035*	0.45 (minimally)	24	7.66 (19.91)	0.0660	0.39 (minimally)
Therapy Setting (0–100)								
Week 12	47	13.81 (23.27)	*< 0.0001*	0.59 (moderately)	24	26.82 (23.40)	*< 0.0001*	1.15 (highly)
Week 24	44	16.61 (22.99)	*< 0.0001*	0.72 (moderately)	24	21.26 (23.40)	*< 0.0001*	0.91 (highly)
Treatment Costs (0–100)								
Week 12	47	11.24 (29.96)	*0.0112*	0.37 (minimally)	24	29.43 (23.64)	*< 0.0001*	1.24 (highly)
Week 24	44	12.51 (29.59)	*0.0058*	0.42 (minimally)	24	25.61 (23.64)	*< 0.0001*	0.92 (highly)

*LQI* Life Quality Index

^a^*p* < 0.05

**Fig. 2:**
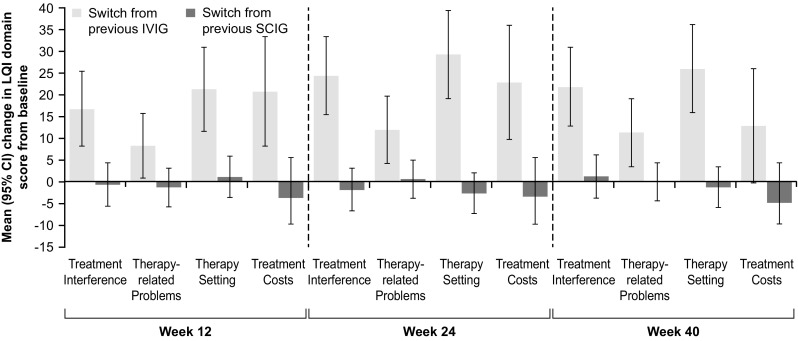
Change from Screening in LQI domain scores by previous IgG therapy in the EU switch study. Data are expressed as mean (95% CI). CI confidence interval, EU European, IgG immunoglobulin G, IVIG intravenous immunoglobulin, LQI Life Quality Index, SCIG subcutaneous immunoglobulin

In the EU switch study, improvement in Therapy-Related Problems continued to Week 40, the final study visit (Fig. 1a). In the JP switch study, as noted earlier, the last visit was in Week 24. In the EU switch study, where patients were allowed to switch from both IVIG or another SCIG to Hizentra®, improvements in the LQI domain scores were largely driven by patients switching from IVIG to SCIG (*n* = 27), rather than patients switching from other SCIG preparations to Hizentra® SCIG (*n* = 19), for whom changes were not statistically significant (Fig. 2).

Changes from Screening in individual LQI item scores in the EU and JP switch studies are shown in Table S1. There were significant improvements for the majority of LQI items at Week 12 and Week 24 in both the EU and JP switch studies.

#### LQI Scores from Pooled Analysis of Switch Studies

**Fig. 3:**
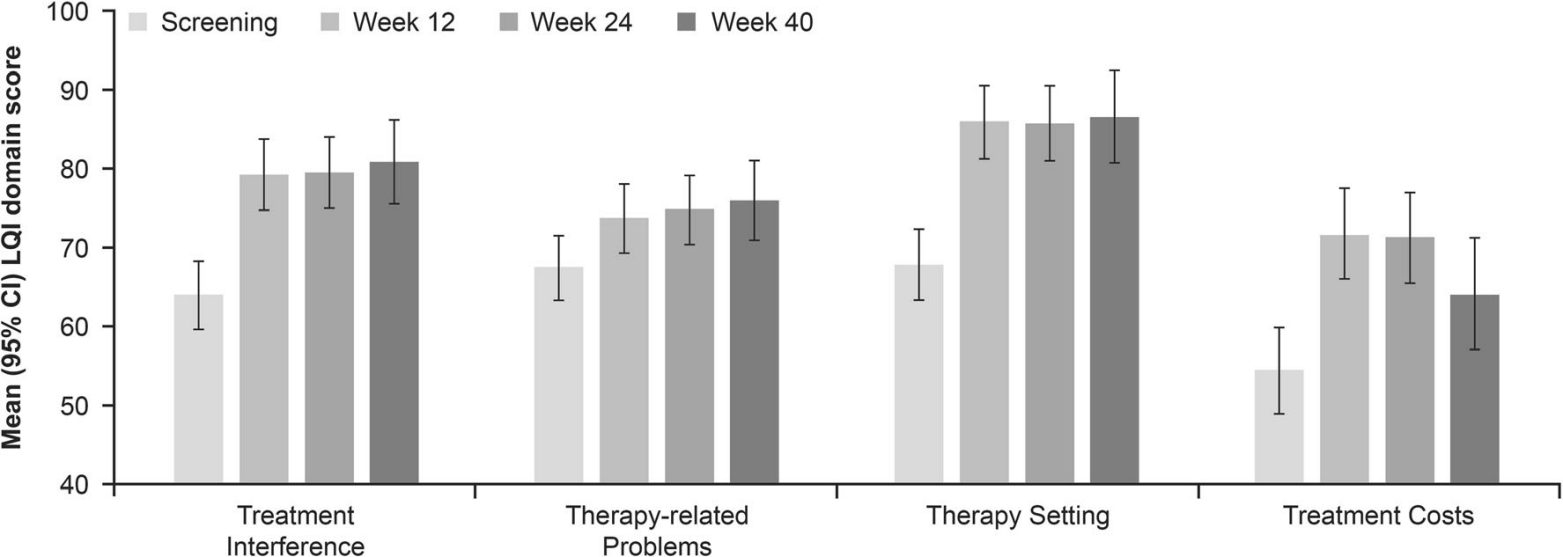
LQI domain scores from the pooled analysis of EU and JP switch studies. Data are expressed as mean (95% CI). CI confidence interval, EU European, JP Japanese, LQI Life Quality Index

Analysis of the pooled data from the EU and JP switch studies showed significant improvements in all LQI domain scores (Treatment Interference, Therapy-Related Problems, Therapy Setting, and Treatment Costs) from Screening. Most improvement occurred between Screening and Week 12, which was sustained at subsequent time points (Weeks 24 and 40, Fig. 3).

**Fig. 4:**
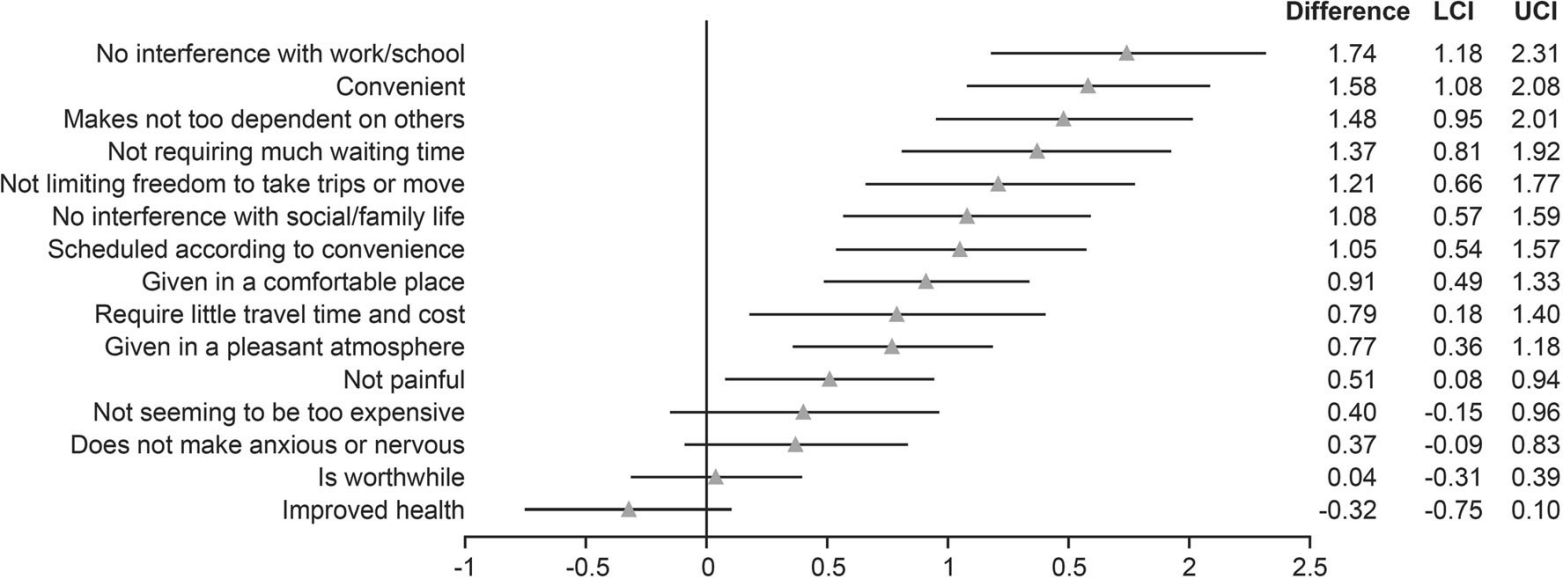
Change from Screening to Week 40 in individual LQI question scores from the pooled data analysis of the EU and JP switch studies. Data are expressed as mean (95% CI). CI confidence interval, EU European, JP Japanese, LCI lower confidence interval, LQI Life Quality Index, UCI upper confidence interval

Changes from Screening to Week 40 in individual LQI item (question) scores from pooled data analysis of the EU and JP switch studies were consistent, with statistically significant improvements in all but four items (Fig. 4).

#### SF-36v2 Scores from Individual Switch Studies

In the EU switch study, there were significant increases in mean SF-36v2 domain scores for Physical Function and General Health from Screening to Week 12 and to Week 24 (Table 3). These improvements were observed also at Week 24 (Table 3). Improvement was also observed for the Role Emotional domain at Weeks 24 and 40; these were minimally-to-moderately meaningful (Table 3). Other domains of the SF-36v2 were not associated with significant or meaningful changes. SF-36v2 was not assessed in the US or JP switch studies.

**Table 3:** SF-36v2 domain scores in the EU switch study

SF-36v2 domain by visit	*N*	Change from Screening, mean (SD)	*p* values of change^a^	Effect size (meaningfulness of change)
Physical Functioning				
Week 12	28	4.66 (8.52)	*0.0049*	0.55 (moderately)
Week 24	25	3.56 (8.40)	*0.0372*	0.42 (minimally)
Role Physical				
Week 12	28	1.53 (17.51)	0.6456	0.087 (not)
Week 24	25	3.71 (17.25)	0.2852	0.22 (minimally)
Bodily Pain				
Week 12	28	2.50 (20.15)	0.5133	0.12 (not)
Week 24	25	6.38 (19.83)	0.1119	0.32 (minimally)
General Health				
Week 12	28	7.20 (17.51)	*0.0326*	0.41 (minimally)
Week 24	25	7.74 (17.23)	*0.0277*	0.45 (minimally)
Vitality				
Week 12	28	4.15 (12.73)	0.0888	0.33 (not)
Week 24	25	− 0.05 (12.55)	0.9854	− 0.004 (not)
Social Functioning				
Week 12	28	− 2.43 (13.57)	0.3463	− 0.18 (not)
Week 24	25	1.23 (13.37)	0.6479	0.092 (not)
Role Emotional				
Week 12	28	1.05 (13.31)	0.6777	0.45 (minimally)
Week 24	25	5.96 (13.11)	*0.0259*	0.47 (minimally)
Mental Health				
Week 12	28	0.32 (12.53)	0.8925	0.026 (not)
Week 24	25	1.25 (12.32)	0.6146	0.11 (not)

*SF-36v2* Short Form 36 version 2

^a^*p* < 0.05

**Fig. 5:**
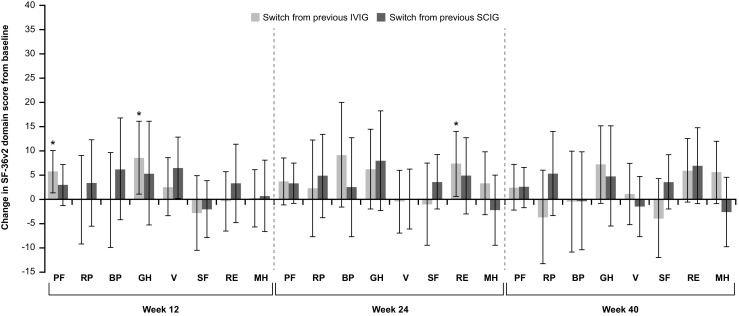
Change from Screening in SF-36v2 domain scores by previous IgG therapy in the EU switch study. Data are expressed as mean (95% CI). BP Bodily Pain, CI confidence interval, EU European, GH General Health, IgG immunoglobulin G, IVIG intravenous immunoglobulin, MH Mental Health, PF Physical Functioning, RE Role-Emotional, RP Role-Physical, SCIG subcutaneous immunoglobulin, SF Social Functioning, SF-36v2 Short Form 36 version 2, V Vitality

In the EU switch study, previous treatment (IVIG vs SCIG) had little impact on change in SF-36v2 scores, although at Week 12, there was a significant improvement in Physical Functioning and Global Health domains in patients switching from IVIG that was not observed in patients switching from SCIG (Fig. 5).

### Maintenance Studies

#### LQI Scores from Individual Maintenance Studies

LQI scores were sustained in the maintenance (follow-up/extension) studies. Mean LQI domain scores in the EU, JP, and US maintenance studies were stable and in one case improved (Fig. 1), suggesting that patient-reported IgG treatment-specific HRQOL was sustained over a long period of time (up to 208 weeks in the combined EU switch and maintenance studies).

#### LQI Scores from Pooled Analysis of Maintenance Studies

**Fig. 6:**
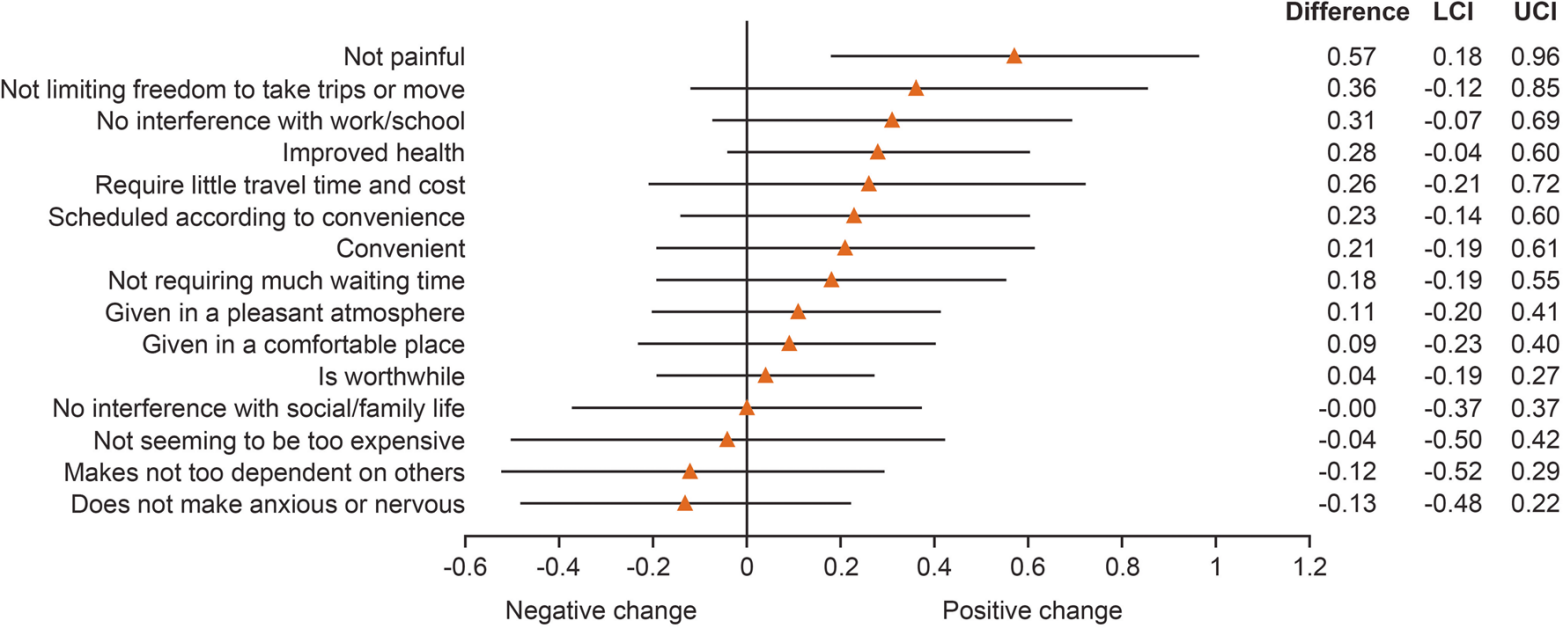
Change from Screening to Month 24 in individual LQI question scores from the pooled data analysis of the EU and US maintenance studies. Data are expressed as mean (95% CI). CI confidence interval, EU European, LCI lower confidence interval, LQI Life Quality Index, UCI upper confidence interval

Analysis of pooled data from the maintenance studies also showed that LQI scores on all four domains were sustained (i.e., no statistically significant longitudinal change) at the follow-up time points; further, there was significant improvement in Therapy-Related Problems at Month 30 and Treatment Costs at Months 6 and 18 (Table S2). Changes from Screening to Month 24 in individual LQI items from the pooled data analysis of the EU and US maintenance studies were positive on 11/15 items, and one even showed a statistically significant improvement (Not Painful; Fig. 6).

#### SF-36v2 Scores from Individual Maintenance Studies

Overall, SF-36v2 domain scores were maintained from Screening to Month 24 in the EU and US maintenance studies; further, there was an improvement in Physical Functioning at Month 6 (+6.53, *p* = 0.03) and Month 12 (+6.04, *p* = 0.04) in the US maintenance study (Table S3).

#### SF-36v2 Scores from Pooled Analysis of Maintenance Studies

**Fig. 7:**
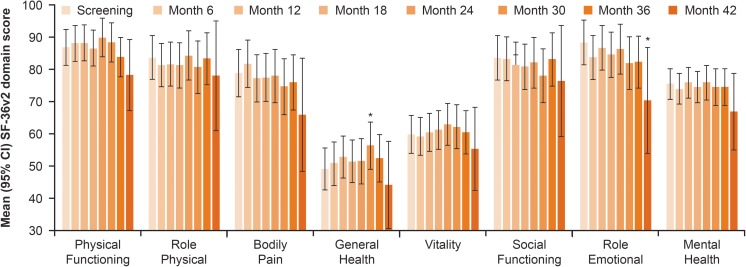
SF-36v2 domain scores from the pooled analysis of the EU and US maintenance studies. Data are expressed as mean (95% CI). *p < 0.05 vs screening visit. CI confidence interval, EU European, SF-36v2 Short Form 36 version 2, US United States

**Fig. 8:**
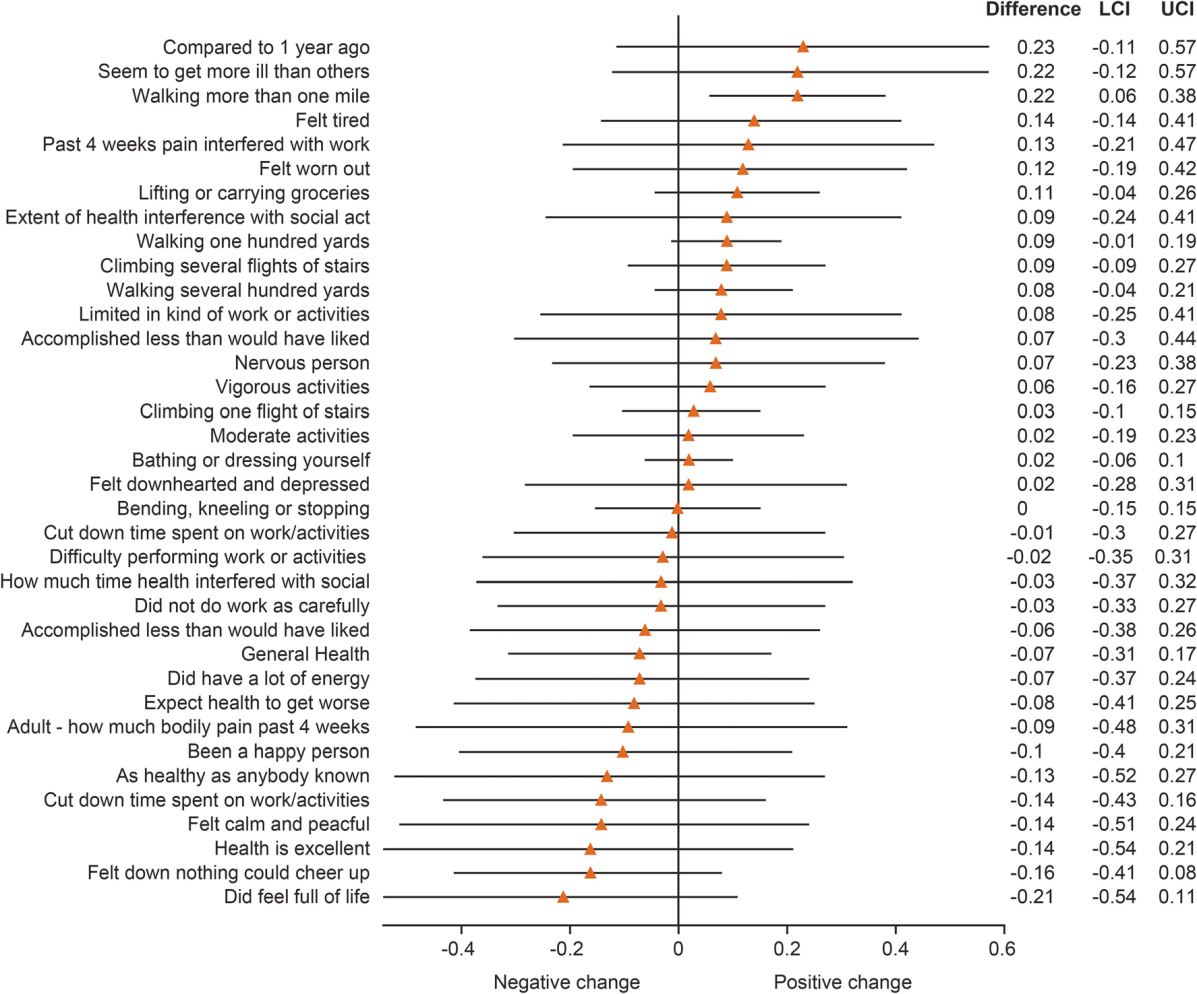
Change from Screening to Month 24 in individual SF-36v2 questions from the pooled data analysis of the EU and US maintenance studies. Data are expressed as mean (95% CI). CI confidence interval, EU European, LCI lower confidence interval, SF-36v2 Short Form 36 version 2, UCI upper confidence interval, US United States

The pooled data analysis of the EU and US maintenance studies showed maintenance of scores on all SF-36v2 domains from Screening to Month 24 of follow-up (Fig. 7). Similar results were observed for changes in individual SF-36v2 questions at Month 24 from the pooled data analysis of these studies (Fig. 8).

## Discussion

This analysis of six Phase 3 clinical studies showed meaningful improvement for several relevant aspects of IgG treatment-specific HRQOL (as measured by the LQI) and health status (as measured by SF-36v2) in patients with PIDD upon switching from IVIG or another SCIG to Hizentra®, which was maintained for up to 3 years. Specifically, in two studies (EU and JP) involving a switch from IVIG to SCIG, LQI and SF-36v2 domain scores improved significantly from initial Screening, as early as Week 12 with sustained improvement through Week 24. In the EU switch study, where patients had an option to switch from either IVIG or another SCIG to Hizentra®, these improvements were almost exclusively driven by patients switching from IVIG to SCIG. In fact, there were no statistically significant changes observed in patients who switched from another SCIG to Hizentra®.

These results on improvement following a switch from IVIG to SCIG are consistent with past studies that have also demonstrated an improvement in health status and IgG-specific HRQOL perceptions, as measured by SF-36 and LQI questionnaires [20, 21, 33, 34]. In one study, significant improvements were seen from baseline to month 10 in the SF-36 domains of Vitality, Mental Health, and Social Functioning in patients switching from IVIG to SCIG [20]. Improvements in other SF-36 domains, including Role Physical, Bodily Pain, Role Emotional, Health Transition, and General Health have also been shown following a switch from IVIG to SCIG [21, 33]. Furthermore, significant improvements in both the LQI summary score and individual LQI domains have also been reported [20, 21, 33, 34]. In our switch studies, the domains most favorably impacted were Therapy Setting and Treatment Interference, and to a smaller extent, Therapy-Related Problems, for the LQI questionnaire, and Physical Function, General Health, and to a lesser extent, Role Emotional for the SF-36v2 questionnaire. Most improvements on the above domains were at or near magnitudes of what is considered at least moderately meaningful change.

Further, while our findings regarding an improvement in outcomes (SF-36v2, LQI) following an IVIG to SCIG switch are consistent with past literature, to our knowledge, ours is the longest follow-up of patients with PIDD who were maintained on SCIG treatment. Generally, following improvement, there were no significant changes in these HRQOL measures during the long-term maintenance studies (conducted in EU, JP, and the US), suggesting that improvements in IgG treatment-specific HRQOL perceptions and patient health status were maintained with long-term Hizentra® treatment (up to 208 weeks). Overall, our results are consistent with a recent study that, in addition to demonstrating improvement with an IVIG to SCIG switch, showed the benefit to be maintained for up to 90 weeks [35].

Findings from our study highlight the multi-dimensional nature of PIDD and its treatment. Evidence from the switch studies demonstrated improvements in domains that reflect patient convenience, comfort, independence, treatment schedule flexibility, pleasantness of treatment setting, and less disruption of daily activities. These improvements are perhaps not surprising, given a change from treatment typically administered intravenously in a healthcare setting to home-based SCIG treatment which offers increased patient flexibility [29]. In addition, although our findings on Treatment Costs based on LQI are not meaningful as a result of the artificial nature of a clinical trial setting, evidence from real-world studies has demonstrated cost savings when switching from IVIG to SCIG for treatment of PIDD [36–38].

The evidence also showed improvements in patient-reported Physical Function and General Health following the IVIG to SCIG switch, consistent with previously reported improvements in Vitality [20, 21]. Improvements in these domains may reflect the impact of stable serum IgG levels characteristic of SCIG treatment, including both higher IgG trough concentrations and reduced peak-to-trough variation achieved through more frequent SCIG treatments, i.e., less wear-off manifested in reduced fatigue [23, 33, 39]. In addition, patients receiving treatment at home may have a reduced exposure to nosocomial infections, further increasing the likelihood of a perceived improvement in general health. Our results therefore support the idea that maintaining stable serum IgG levels translates into improvements in physical function, and moreover, overall general health.

Consistent with the multidimensional nature of PIDD and its treatment, we observed no improvement in some domains of patient-reported outcomes. The Mental Health summary score as measured by SF-36v2 did not change after switching to SCIG. Similarly, the Treatment Costs domain on the LQI was generally not impacted. At the item level, there was lack of improvement with an IVIG to SCIG switch on the LQI items of *Improved Health*, *Treatment Being Worthwhile*, and *Does Not Make Anxious or Nervous*. Findings such as these, especially on the overarching LQI item *Improved Health*, arguably serve as reminders that PIDD is a chronic condition and may continue to exact a toll in the long run, unaffected by a switch in treatment modality. Further, domains such as emotional status are less likely to be impacted given that they are likely confounded by additional extraneous influences (not explored in these studies), consistent with the Wilson Cleary classification of health outcomes into those that are more immediately impacted by interventions (in this case, a switch from IVIG to SCIG) and those that are likely to be confounded by other factors [40]. Accordingly, it is also not surprising that while a switch in modality from IVIG to SCIG was manifested in an immediate improvement in domains that measured convenience aspects, but not so on domains and items relating to perceptions of intervention cost, as all study medication costs were covered by the study sponsor. However, in clinical practice, these factors are likely to impact patients variously, based on their healthcare system dynamics. Absence of improvement on the item *Does Not Make Them Anxious or Nervous* following a change to SCIG in both the switch and long-term maintenance phases may highlight the need for physicians to carefully consider those who may not be good candidates for self-infusion by encouraging shared decision making in consultation with the patient.

Finally, our findings on fatigue were somewhat mixed. In past research, patients with primary antibody deficiency have been shown to have higher fatigue levels (25.9%; 95% CI 23.7–28.3) compared with the general population (6–7.5%) [41, 42] and to patients with PIDD who had a condition different from primary antibody deficiency (6.3%; 95% CI 4.9–8.2) [43]. Moreover, patients with common variable immune deficiency, which constituted the large majority of the population in our studies, have been shown to have the highest fatigue levels of all patients with PAD [43]. Fatigue levels have also been shown to be similar regardless whether patients received IVIG or SCIG [43]. In our studies, we assessed Vitality, i.e., the obverse of fatigue, as part of the SF-36v2 and found scores to be uniformly lower (worse) than on other domains, consistent with past work. We also found only marginal improvements in Vitality in the switch studies, which were nevertheless maintained in the longer term, again potentially reflective of the chronic nature of disease and frequency of treatment.

### Strengths and Limitations of This Analysis

This is the first analysis of pooled data from two switch and four maintenance (follow-up/extension) studies evaluating patient-reported HRQOL outcomes both after a switch from IVIG to SCIG treatment and during long-term SCIG maintenance treatment, in patients with PIDD. Further, this study is unique in reporting not just outcomes by domain, but also improvements on each of the 15 LQI items and 36 SF-36v2 items. Recently, there has been debate over the utility of aggregation scores from ordinal scales to arrive at summary measures, as is often reported [44]. Indeed, the developers of the SF-36 questionnaire have stated that it is not appropriate to combine all domains to produce one overall score [44]. While advanced psychometric evaluation of the measurement and aggregation properties of the items based on modern item response theory [45, 46] was outside the scope of this paper, at least by presenting outcomes for individual items, in addition to domains, we hope this paper provides a more transparent profile of the impact of IgG therapy on HRQOL.

Limitations include the different time points of data collection across studies, allowing for comparison and pooling of data at selected time points only, and the fact that completed questionnaires were not returned by all patients at each time point. Furthermore, an active comparator cohort or placebo cohort were not included into design of these studies. Finally, many of the factors that may have confounded HRQOL, including complexities with self-administration (such as vial sizes and/or numbers of times the vials needed to be switched, and need to replace the pump) as well as the influence of any improvements in serum trough IgG levels were not specifically analyzed for this report.

## Conclusions

The results from the switch (pivotal) Hizentra® studies where HRQOL was assessed showed that switching patients from IVIG to SCIG improves patient self-reported health status and IgG-specific HRQOL perception. The maintenance (extension) studies generally showed no deterioration of the improved health status achieved when switching from IVIG to SCIG treatment with Hizentra® over a long follow-up period.

## Electronic supplementary material

ESM 1 (DOCX 281 kb)

ESM 2 (JPG 1142 kb)

## References

[1] Picard C, Al-Herz W, Bousfiha A, Casanova JL, Chatila T, Conley ME (2015). Primary immunodeficiency diseases: an update on the classification from the International Union of Immunological Societies Expert Committee for Primary Immunodeficiency 2015. J Clin Immunol.

[2] Wood P, Stanworth S, Burton J, Jones A, Peckham DG, Green T, Hyde C, Chapel H, the UK Primary Immunodeficiency Network (2007). Recognition, clinical diagnosis and management of patients with primary antibody deficiencies: a systematic review. Clin Exp Immunol.

[3] Boyle JM, Buckley RH (2007). Population prevalence of diagnosed primary immunodeficiency diseases in the United States. J Clin Immunol.

[4] Immune Deficiency Foundation. Primary immunodeficiency disease in America. The third national survey of patients 2007. 2007. Available from: https://primaryimmune.org/wp-content/uploads/2011/04/Primary-Immunodeficiency-Diseases-in-America-2007The-Third-National-Survey-of-Patients.pdf.

[5] Kuburovic NB, Pasic S, Susic G, Stevanovic D, Kuburovic V, Zdravkovic S, Janicijevic Petrovic M, Pekmezovic T (2014). Health-related quality of life, anxiety, and depressive symptoms in children with primary immunodeficiencies. Patient Prefer Adherence.

[6] Mozaffari H, Pourpak Z, Pourseyed S, Moin M, Farhoodi A, Aghamohammadi A (2006). Health-related quality of life in primary immune deficient patients. Iran J Allergy Asthma Immunol.

[7] Soresina A, Nacinovich R, Bomba M, Cassani M, Molinaro A, Sciotto A (2009). The quality of life of children and adolescents with X-linked agammaglobulinemia. J Clin Immunol.

[8] Tabolli S, Giannantoni P, Pulvirenti F, La Marra F, Granata G, Milito C (2014). Longitudinal study on health-related quality of life in a cohort of 96 patients with common variable immune deficiencies. Front Immunol.

[9] Zebracki K, Palermo TM, Hostoffer R, Duff K, Drotar D (2004). Health-related quality of life of children with primary immunodeficiency disease: a comparison study. Ann Allergy Asthma Immunol.

[10] Busse PJ, Razvi S, Cunningham-Rundles C (2002). Efficacy of intravenous immunoglobulin in the prevention of pneumonia in patients with common variable immunodeficiency. J Allergy Clin Immunol.

[11] Quartier P, Debré M, De Blic J, de Sauverzac R, Sayegh N, Jabado N (1999). Early and prolonged intravenous immunoglobulin replacement therapy in childhood agammaglobulinemia: a retrospective survey of 31 patients. J Pediatr.

[12] Abdou NI, Greenwell CA, Mehta R, Narra M, Hester JD, Halsey JF (2009). Efficacy of intravenous gammaglobulin for immunoglobulin G subclass and/or antibody deficiency in adults. Int Arch Allergy Immunol.

[13] Berger M (2008). Principles of and advances in immunoglobulin replacement therapy for primary immunodeficiency. Immunol Allergy Clin N Am.

[14] Gathmann B, Grimbacher B, Beaute J, Dudoit Y, Mahlaoui N, Fischer A (2009). The European internet-based patient and research database for primary immunodeficiencies: results 2006-2008. Clin Exp Immunol.

[15] Jolles S, Borte M, Nelson RP, Rojavin M, Bexon M, Lawo JP, Wasserman RL (2014). Long-term efficacy, safety, and tolerability of Hizentra(R) for treatment of primary immunodeficiency disease. Clin Immunol.

[16] Jolles S, Jordan SC, Orange JS, van Schaik IN (2014). Immunoglobulins: current understanding and future directions. Clin Exp Immunol.

[17] Jiang F, Torgerson TR, Ayars AG (2015). Health-related quality of life in patients with primary immunodeficiency disease. Allergy Asthma Clin Immunol.

[18] Ochs HD, Gupta S, Kiessling P, Nicolay U, Berger M (2006). Safety and efficacy of self-administered subcutaneous immunoglobulin in patients with primary immunodeficiency diseases. J Clin Immunol.

[19] Wasserman RL, Melamed I, Nelson RP, Knutsen AP, Fasano MB, Stein MR (2012). Pharmacokinetics of subcutaneous IgPro20 in patients with primary immunodeficiency. Clin Pharmacokinet.

[20] Gardulf A, Nicolay U, Math D, Asensio O, Bernatowska E, Bock A (2004). Children and adults with primary antibody deficiencies gain quality of life by subcutaneous IgG self-infusions at home. J Allergy Clin Immunol.

[21] Nicolay U, Kiessling P, Berger M, Gupta S, Yel L, Roifman CM, Gardulf A, Eichmann F, Haag S, Massion C, Ochs HD (2006). Health-related quality of life and treatment satisfaction in North American patients with primary immunedeficiency diseases receiving subcutaneous IgG self-infusions at home. J Clin Immunol.

[22] Landersdorfer C, Bexon M, Edelman J, Rojavin M, Kirkpatrick C, Lu J (2013). Pharmacokinetic modeling and simulation of biweekly subcutaneous immunoglobulin dosing in primary immunodeficiency. Postgrad Med.

[23] Sidhu J, Rojavin MA, Pfister M, Edelman J (2014). Enhancing patient flexibility of subcutaneous immunoglobulin G dosing: pharmacokinetic outcomes of various maintenance and loading regimens in the treatment of primary immunodeficiency. Biol Ther.

[24] Ponsford M, Carne E, Kingdon C, Joyce C, Price C, Williams C, el-Shanawany T, Williams P, Jolles S (2015). Facilitated subcutaneous immunoglobulin (fSCIg) therapy—practical considerations. Clin Exp Immunol.

[25] Stein MR, Nelson RP, Church JA, Wasserman RL, Borte M, Vermylen C, Bichler J, for the IgPro10 in PID study group (2009). Safety and efficacy of Privigen^®^, a novel 10% liquid immunoglobulin preparation for intravenous use, in patients with primary immunodeficiencies. J Clin Immunol.

[26] Jolles S, Sleasman JW (2011). Subcutaneous immunoglobulin replacement therapy with Hizentra^®^, the first 20% SCIG preparation: a practical approach. Adv Ther.

[27] Wasserman RL (2014). Hizentra for the treatment of primary immunodeficiency. Expert Rev Clin Immunol.

[28] Hagan JB, Fasano MB, Spector S, Wasserman RL, Melamed I, Rojavin MA, Zenker O, Orange JS (2010). Efficacy and safety of a new 20% immunoglobulin preparation for subcutaneous administration, IgPro20, in patients with primary immunodeficiency. J Clin Immunol.

[29] Jolles S, Bernatowska E, de Gracia J, Borte M, Cristea V, Peter HH, Belohradsky BH, Wahn V, Neufang-Hüber J, Zenker O, Grimbacher B (2011). Efficacy and safety of Hizentra^®^ in patients with primary immunodeficiency after a dose-equivalent switch from intravenous or subcutaneous replacement therapy. Clin Immunol.

[30] Kanegane H, Imai K, Yamada M, Takada H, Nonoyama S, Ariga T (2014). Efficacy and safety of IgPro20, a subcutaneous immunoglobulin, in Japanese patients with primary immunodeficiency diseases. J Clin Immunol.

[31] Daly P, Evans J, Kobayashi R, Kobayashi A, Ochs H, Fischer S, Pirofsky B, Sprouse C (1991). Home-based immunoglobulin infusion therapy: quality of life and patient health perceptions. Ann Allergy.

[32] Cohen J (1988). Statistical power analysis for the behavioral sciences (revised edition).

[33] Gardulf A, Borte M, Ochs HD, Nicolay U (2008). Prognostic factors for health-related quality of life in adults and children with primary antibody deficiencies receiving SCIG home therapy. Clin Immunol.

[34] Igarashi A, Kanegane H, Kobayashi M, Miyawaki T, Tsutani K (2014). Cost-minimization analysis of IgPro20, a subcutaneous immunoglobulin, in Japanese patients with primary immunodeficiency. Clin Ther.

[35] Suez D, Stein M, Gupta S, Hussain I, Melamed I, Paris K, Darter A, Bourgeois C, Fritsch S, Leibl H, McCoy B, Gelmont D, Yel L (2016). Efficacy, safety, and pharmacokinetics of a novel human immune globulin subcutaneous, 20% in patients with primary immunodeficiency diseases in North America. J Clin Immunol.

[36] Beaute J, Levy P, Millet V, Debre M, Dudoit Y, Le Mignot L (2010). Economic evaluation of immunoglobulin replacement in patients with primary antibody deficiencies. Clin Exp Immunol.

[37] Martin A, Lavoie L, Goetghebeur M, Schellenberg R (2013). Economic benefits of subcutaneous rapid push versus intravenous immunoglobulin infusion therapy in adult patients with primary immune deficiency. Transfus Med.

[38] Perraudin C, Bourdin A, Spertini F, Berger J, Bugnon O (2016). Switching patients to home-based subcutaneous immunoglobulin: an economic evaluation of an interprofessional drug therapy management program. J Clin Immunol.

[39] Waniewski J, Gardulf A, Hammarström L (1994). Bioavailability of gamma-globulin after subcutaneous infusions in patients with common variable immunodeficiency. J Clin Immunol.

[40] Wilson IB, Cleary PD (1995). Linking clinical variables with health-related quality of life. A conceptual model of patient outcomes. JAMA.

[41] Afari N, Buchwald D (2003). Chronic fatigue syndrome: a review. Am J Psychiatry.

[42] Walker EA, Katon WJ, Jemelka RP (1993). Psychiatric disorders and medical care utilization among people in the general population who report fatigue. J Gen Intern Med.

[43] Hajjar J, Guffey D, Minard CG, Orange JS (2017). Increased incidence of fatigue in patients with primary immunodeficiency disorders: prevalence and associations within the US Immunodeficiency Network registry. J Clin Immunol.

[44] Lins L, Carvalho FM. SF-36 total score as a single measure of health-related quality of life: Scoping review. SAGE Open Med. 2016;4. 10.1177/2050312116671725 PMC5052926 27757230

[45] Fries J, Bruce B, Cella D. The promise of PROMIS: Using item response theory to improve assessment of patient-reported outcomes. 2005. S53–7 p. 16273785

[46] Hays RD, Morales LS, Reise SP (2000). Item response theory and health outcomes measurement in the 21st century. Med Care.

