# Supplementary material for: Treatment Satisfaction with Subcutaneous Immunoglobulin Replacement Therapy in Patients with Primary Immunodeficiency: a Pooled Analysis of Six Hizentra® Studies
Source: J Clin Immunol. 2018 Nov 21;38(8):886–97. doi: 10.1007/s10875-018-0562-3 (PMC6292975; doi:10.1007/s10875-018-0562-3)
Supplement: Supplementary file 1 — (DOCX 281 kb) [file 10875_2018_562_MOESM1_ESM.docx]

# Supplementary Material

## Supplementary Figure Legend

**Fig. S1 Questionnaire completion time points.** In the JP and EU switch (pivotal) studies and the JP maintenance (follow-up) studies, the HRQOL questionnaires were completed every 12 weeks and at the final visit. In the EU maintenance (extension) study), the HRQOL questionnaires were completed every 6 months up to 42 months. In the US maintenance (extension) study, the LQI questionnaire was completed every 60 weeks and the SF-36v2 questionnaire every 24 weeks. HRQOL questionnaires were not completed in the second JP maintenance (extension) study or the US switch (pivotal) study. EU, European; LQI, Life Quality Index; US, United States; SF-36v2, Short Form-36 version 2

# Supplementary Material

## Table S1. Descriptive statistics for individual LQI question scores in the EU and JP switch studies

| **LQI questions by switch study** | **Screening**  **Mean and 95% CI* Week 12**  **Week 24** | **Difference from Screening (95% CI of difference)** | **P-values**  **of difference to Screening** |
| --- | --- | --- | --- |
| **Convenient** | | | |
| EU |  | Week 12: 1.11 (0.63; 1.60)  Week 24: 1.34 (0.85; 1.83) | Week 12: **<0.0001**  Week 24: **<0.0001** |
| JP |  | Week 12: 2.38 (1.52; 3.24)  Week 24: 2.34 (1.50; 3.19) | Week 12: **<0.0001**  Week 24: **<0.0001** |
| **Not painful** | | | |
| EU |  | Week 12: 0.40 (-0.03; 0.84)  Week 24: 0.48 (0.03; 0.92) | Week 12: 0.0709  Week 24: **0.0366** |
| JP |  | Week 12: -0.11 (-0.76; 0.53)  Week 24: -0.24 (-0.88; 0.40) | Week 12: 0.7238  Week 24: 0.4590 |
| **Improved health** | | | |
| EU |  | Week 12: -0.73 (-1.15; -0.30)  Week 24: -0.31 (-0.74; 0.13) | Week 12: **0.0009**  Week 24: 0.1655 |
| JP |  | Week 12: -0.06 (-0.76; 0.64)  Week 24: -0.35 (-1.05; 0.35) | Week 12: 0.8717  Week 24: 0.3221 |
| **No interference with social/family life** | | | |
| EU |  | Week 12: 0.38 (-0.15; 0.91)  Week 24: 0.81 (0.26; 1.35) | Week 12: 0.1614  Week 24: **0.0041** |
| JP |  | Week 12: 1.66 (0.96; 2.37)  Week 24: 1.29 (0.58; 1.99) | Week 12: **<0.0001**  Week 24: **0.0006** |
| **No interference with work/school** | | | |
| EU |  | Week 12: 1.31 (0.70; 1.91)  Week 24: 1.67 (1.05; 2.28) | Week 12: **<0.0001**  Week 24: **<0.0001** |
| JP |  | Week 12: 2.59 (1.83; 3.36)  Week 24: 1.76 (0.99; 2.53) | Week 12: **<0.0001**  Week 24: **<0.0001** |
| **Given in a comfortable place** | | | |
| EU |  | Week 12: 0.59 (0.18; 1.01)  Week 24: 0.70 (0.28; 1.12) | Week 12: **0.0050**  Week 24: **0.0012** |
| JP |  | Week 12: 1.15 (0.44; 1.87)  Week 24: 1.07 (0.36; 1.79) | Week 12: **0.0022**  Week 24: **0.0042** |
| **Not requiring much waiting time** | | | |
| EU |  | Week 12: 0.64 (0.13; 1.15)  Week 24: 0.84 (0.32; 1.36) | Week 12: **0.0143**  Week 24: **0.0017** |
| JP |  | Week 12: 3.07 (2.17; 3.98)  Week 24: 2.91 (2.00; 3.81) | Week 12: **<0.0001**  Week 24: **<0.0001** |
| **Given in a pleasant atmosphere** | | | |
| EU |  | Week 12: 0.58 (0.19; 0.97)  Week 24: 0.61 (0.21; 1.01) | Week 12: **0.0042**  Week 24: **0.0029** |
| JP |  | Week 12: 1.17 (0.40; 1.93)  Week 24: 1.00 (0.24; 1.75) | Week 12: **0.0035**  Week 24: **0.0107** |
| **Is worthwhile** | | | |
| EU |  | Week 12: -0.11 (-0.45; 0.23)  Week 24: -0.13 (-0.48; 0.22) | Week 12: 0.5366  Week 24: 0.4602 |
| JP |  | Week 12: 0.39 (-0.21; 1.00)  Week 24: 0.39 (-0.21; 1.00) | Week 12: 0.2001  Week 24: 0.2001 |
| **Does not make anxious or nervous** | | | |
| EU |  | Week 12: 0.34 (-0.08; 0.77)  Week 24: 0.32 (-0.11; 0.75) | Week 12: 0.1132  Week 24: 0.1473 |
| JP |  | Week 12: 0.16 (-0.72; 1.05)  Week 24: 0.08 (-0.80; 0.97) | Week 12: 0.7108  Week 24: 0.8552 |
| **Not seeming to be too expensive** | | | |
| EU |  | Week 12: 0.54 (-0.09; 1.16)  Week 24: 0.60 (-0.02; 1.22) | Week 12: **0.0900**  Week 24: 0.0575 |
| JP |  | Week 12: 1.57 (0.87; 2.26)  Week 24: 1.28 (0.58; 1.97) | Week 12: **<0.0001**  Week 24: **0.0006** |
| **Does not make me too dependent on** **others** | | | |
| EU |  | Week 12: 1.22 (0.67; 1.76)  Week 24: 1.41 (0.86; 1.96) | Week 12: **<0.0001**  Week 24: **<0.0001** |
| JP |  | Week 12: 2.03 (1.18; 2.89)  Week 24: 1.78 (0.93; 2.64) | Week 12: **<0.0001**  Week 24: **0.0001** |
| **Require little travel time and cost** | | | |
| EU |  | Week 12: 0.67 (0.01; 1.32)  Week 24: 0.88 (0.21; 1.56) | Week 12: **0.0476**  Week 24: **0.0102** |
| JP |  | Week 12: 1.96 (1.17; 2.76)  Week 24: 1.80 (1.00; 2.59) | Week 12: **<0.0001**  Week 24: **<0.0001** |
| **Not limiting freedom to take trips or** **move** | | | |
| EU |  | Week 12: 0.87 (0.31; 1.43)  Week 24: 1.22 (0.65; 1.79) | Week 12: **0.0025**  Week 24: **<0.0001** |
| JP |  | Week 12: 1.20 (0.31; 2.10)  Week 24: 0.25 (-0.65; 1.15) | Week 12: **0.0093**  Week 24: 0.5817 |
| **Scheduled according to convenience** | | | |
| EU |  | Week 12: 0.58 (0.03; 1.13)  Week 24: 0.78 (0.22; 1.34) | Week 12: **0.0374**  Week 24: **0.0067** |
| JP |  | Week 12: 1.12 (0.38; 1.86)  Week 24: 0.55 (-0.20; 1.30) | Week 12: **0.0039**  Week 24: 0.1471 |
| *On the scale from 1 (worst outcome) to 7 (best outcome); ^†^p<0.05 vs screening. | | | |

##

## Table S2. Descriptive statistics for LQI domain scores from pooled data from the EU and US maintenance and JP maintenance studies*

| **LQI domains by visit** | **N** | **Mean (95% CI)** | **Difference  (95% CI of difference)** | **p-values of difference** |
| --- | --- | --- | --- | --- |
| **Treatment Interference (0-100)** |  |  |  |  |
| Screening | 80 | 80.77 (77.19; 84.36) | – | – |
| Month 6 | 60 | 81.05 (77.07; 85.03) | 0.28 (-3.52; 4.08) | 0.8864 |
| Month 12 | 38 | 84.86 (80.18; 89.54) | 4.08 (-0.45; 8.61) | 0.0772 |
| Month 18 | 37 | 82.07 (77.35; 86.80) | 1.30 (-3.28; 5.87) | 0.5773 |
| Month 24 | 37 | 82.60 (77.87; 87.32) | 1.82 (-2.75; 6.40) | 0.4336 |
| Month 30 | 37 | 84.40 (79.68; 89.12) | 3.62 (-0.95; 8.20) | 0.1200 |
| **Therapy-Related Problems (0-100)** |  |  |  |  |
| Screening | 80 | 74.96 (71.21; 78.70) | – | – |
| Month 6 | 60 | 74.58 (70.46; 78.69) | -0.38 (-4.01; 3.25) | 0.8369 |
| Month 12 | 38 | 78.31 (73.57; 83.06) | 3.36 (-0.98; 7.69) | 0.1287 |
| Month 18 | 37 | 78.35 (73.57; 83.14) | 3.40 (-0.98; 7.78) | 0.1279 |
| Month 24 | 37 | 78.47 (73.68; 83.25) | 3.51 (-0.87; 7.89) | 0.1158 |
| Month 30 | 37 | 79.48 (74.69; 84.26) | 4.52 (0.14; 8.90) | **0.0430** |
| **Therapy Setting (0-100)** |  |  |  |  |
| Screening | 80 | 86.17 (82.53; 89.81) | – | – |
| Month 6 | 60 | 86.42 (82.36; 90.47) | 0.25 (-3.73; 4.22) | 0.9027 |
| Month 12 | 38 | 90.67 (85.87; 95.47) | 4.50 (-0.23; 9.23) | 0.0620 |
| Month 18 | 37 | 87.82 (82.98; 92.67) | 1.65 (-3.13; 6.43) | 0.4966 |
| Month 24 | 37 | 88.87 (84.03; 93.72) | 2.70 (-2.07; 7.48) | 0.2663 |
| Month 30 | 37 | 89.62 (84.78; 94.47) | 3.45 (-1.32; 8.23) | 0.1558 |
| **Treatment Costs (0-100)** |  |  |  |  |
| Screening | 80 | 72.06 (67.40; 76.71) | – | – |
| Month 6 | 60 | 77.82 (72.61; 83.03) | 5.76 (0.55; 10.98) | **0.0303** |
| Month 12 | 38 | 77.85 (71.65; 84.04) | 5.79 (-0.41; 11.99) | 0.0671 |
| Month 18 | 37 | 78.36 (72.10; 84.61) | 6.30 (0.04; 12.56) | **0.0487** |
| Month 24 | 37 | 73.85 (67.59; 80.11) | 1.79 (-4.47; 8.06) | 0.5730 |
| Month 30 | 37 | 74.75 (68.49; 81.01) | 2.70 (-3.57; 8.96) | 0.3975 |

*Screening and Month 6 time points include data from EU and US maintenance and JP maintenance studies, Month 12, 18, 24 and 30 time points include data from the EU and US maintenance studies.

## Table S3. Descriptive statistics for SF-36v2 domains in EU and US maintenance studies

|  | **EU maintenance** | | | |  | | **US maintenance** | | |
| --- | --- | --- | --- | --- | --- | --- | --- | --- | --- |
| **SF-36v2 domains by visit** | **N** | **Mean (95%)** | **Difference  from Screening  (95% CI of difference)** | **p-values of difference** | **N** | **Mean (95% CI)** | | **Difference  from screening  (95% CI of difference)** | **p-values of difference** |
| **Physical Functioning (0-100)** |  |  |  |  |  |  | |  |  |
| Screening | 22 | 92.46 (86.37; 98.55) | **–** | **–** | 17 | 79.78 (70.11; 89.44) | | **–** | **–** |
| Month 6 | 20 | 89.31 (83.12; 95.50) | -3.15 (-8.05; 1.76) | 0.2061 | 19 | 86.30 (76.75; 95.86) | | 6.53 (0.76; 12.30) | **0.0275** |
| Month 12 | 20 | 90.26 (84.06; 96.46) | -2.20 (-7.08; 2.68) | 0.3742 | 18 | 85.82 (76.19; 95.44) | | 6.04 (0.15; 11.93) | **0.0448** |
| Month 18 | 20 | 91.30 (85.10; 97.50) | -1.16 (-6.08; 3.77) | 0.6427 | 16 | 80.88 (71.12; 90.64) | | 1.11 (-4.95; 7.16) | 0.7156 |
| Month 24 | 20 | 93.47 (87.26; 99.68) | 1.01 (-3.88; 5.90) | 0.6839 | 4 | 84.47 (71.79; 97.15) | | 4.69 (-5.36; 14.74) | 0.3529 |
| **Role Physical (0-100)** |  |  |  |  |  |  | |  |  |
| Screening | 22 | 85.07 (76.73; 93.41) | **–** | **–** | 17 | 82.17 (70.50; 93.85) | | **–** | **–** |
| Month 6 | 20 | 84.91 (76.40; 93.43) | -0.16 (-7.67; 7.35) | 0.9669 | 19 | 77.05 (65.68; 88.42) | | -5.13 (-15.74; 5.49) | 0.3368 |
| Month 12 | 20 | 84.41 (75.87; 92.95) | -0.66 (-8.14; 6.81) | 0.8605 | 18 | 78.17 (66.63; 89.71) | | -4.00 (-14.82; 6.82) | 0.4613 |
| Month 18 | 19 | 87.10 (78.46; 95.73) | 2.02 (-5.63; 9.68) | 0.6014 | 16 | 74.25 (62.33; 86.16) | | -7.93 (-19.06; 3.21) | 0.1590 |
| Month 24 | 20 | 89.01 (80.47; 97.56) | 3.94 (-3.54; 11.42) | 0.2988 | 4 | 71.95 (52.91; 90.99) | | -10.22 (-28.68; 8.23) | 0.2712 |
| **Bodily Pain (0-100)** |  |  |  |  |  |  | |  |  |
| Screening | 22 | 83.27 (73.90; 92.64) | **–** | **–** | 17 | 73.54 (62.05; 85.02) | | **–** | **–** |
| Month 6 | 21 | 85.98 (76.54; 95.43) | 2.71 (-5.31; 10.73) | 0.5042 | 19 | 76.59 (65.39; 87.79) | | 3.05 (-7.16; 13.27) | 0.5508 |
| Month 12 | 20 | 82.45 (72.88; 92.03) | -0.82 (-8.92; 7.29) | 0.8421 | 18 | 70.93 (59.58; 82.29) | | -2.60 (-13.02; 7.81) | 0.6176 |
| Month 18 | 21 | 84.55 (75.08; 94.01) | 1.27 (-6.78; 9.32) | 0.7545 | 16 | 68.74 (57.03; 80.45) | | -4.80 (-15.51; 5.92) | 0.3731 |
| Month 24 | 20 | 84.61 (75.03; 94.19) | 1.34 (-6.78; 9.45) | 0.7444 | 4 | 62.50 (44.02; 80.98) | | -11.04 (-28.80; 6.72) | 0.2178 |
| **General Health (0-100)** |  |  |  |  |  |  | |  |  |
| Screening | 22 | 49.45 (40.52; 58.38) | **–** | **–** | 17 | 48.83 (38.86; 58.80) | | **–** | **–** |
| Month 6 | 21 | 52.65 (43.67; 61.62) | 3.19 (-2.70; 9.08) | 0.2853 | 19 | 48.90 (39.12; 58.68) | | 0.07 (-7.68; 7.82) | 0.9860 |
| Month 12 | 20 | 54.19 (45.14; 63.24) | 4.74 (-1.21; 10.69) | 0.1176 | 18 | 51.41 (41.52; 61.29) | | 2.58 (-5.33; 10.48) | 0.5159 |
| Month 18 | 21 | 52.15 (43.16; 61.13) | 2.69 (-3.23; 8.61) | 0.3697 | 16 | 50.83 (40.70; 60.95) | | 2.00 (-6.14; 10.13) | 0.6241 |
| Month 24 | 20 | 52.59 (43.54; 61.64) | 3.14 (-2.82; 9.10) | 0.2995 | 4 | 49.85 (35.00; 64.70) | | 1.02 (-12.47; 14.51) | 0.8797 |
| **Vitality (0-100)** |  |  |  |  |  |  | |  |  |
| Screening | 22 | 65.14 (57.62; 72.65) | **–** | **–** | 17 | 53.97 (45.21; 62.72) | | **–** | **–** |
| Month 6 | 21 | 67.54 (59.99; 75.10) | 2.40 (-2.89; 7.70) | 0.3705 | 19 | 49.41 (40.91; 57.90) | | -4.56 (-13.04; 3.93) | 0.2862 |
| Month 12 | 20 | 66.80 (59.18; 74.43) | 1.66 (-3.69; 7.02) | 0.5392 | 18 | 52.91 (44.27; 61.54) | | -1.06 (-9.71; 7.59) | 0.8066 |
| Month 18 | 21 | 66.71 (59.15; 74.28) | 1.58 (-3.74; 6.90) | 0.5584 | 16 | 54.79 (45.84; 63.75) | | 0.83 (-8.07; 9.73) | 0.8523 |
| Month 24 | 20 | 69.28 (61.65; 76.91) | 4.14 (-1.22; 9.50) | 0.1286 | 4 | 54.01 (39.15; 68.86) | | 0.04 (-14.71; 14.79) | 0.9957 |
| **Social Functioning (0-100)** |  |  |  |  |  |  | |  |  |
| Screening | 22 | 89.31 (81.68; 96.95) | **–** | **–** | 17 | 76.86 (64.72; 89.01) | | **–** | **–** |
| Month 6 | 21 | 91.21 (83.51; 98.92) | 1.90 (-4.99; 8.80) | 0.5861 | 19 | 74.25 (62.51; 86.00) | | -2.61 (-14.84; 9.63) | 0.6703 |
| Month 12 | 20 | 86.44 (78.62; 94.26) | -2.87 (-9.84; 4.09) | 0.4159 | 18 | 75.79 (63.83; 87.75) | | -1.07 (-13.53; 11.39) | 0.8635 |
| Month 18 | 21 | 88.02 (80.30; 95.75) | -1.29 (-8.21; 5.63) | 0.7130 | 16 | 72.89 (60.46; 85.32) | | -3.98 (-16.81; 8.86) | 0.5366 |
| Month 24 | 20 | 88.83 (81.01; 96.66) | -0.48 (-7.45; 6.50) | 0.8921 | 4 | 71.10 (49.99; 92.21) | | -5.77 (-27.00; 15.47) | 0.5880 |
| **Role Emotional (0-100)** |  |  |  |  |  |  | |  |  |
| Screening | 22 | 91.54 (83.61; 99.46) | **–** | **–** | 17 | 84.85 (72.67; 97.03) | | **–** | **–** |
| Month 6 | 21 | 90.73 (82.74; 98.71) | -0.81 (-7.24; 5.62) | 0.8034 | 19 | 75.84 (64.02; 87.67) | | -9.00 (-20.66; 2.65) | 0.1270 |
| Month 12 | 20 | 91.97 (83.88; 100.05) | 0.43 (-6.06; 6.93) | 0.8955 | 18 | 80.81 (68.79; 92.83) | | -4.04 (-15.92; 7.84) | 0.4976 |
| Month 18 | 21 | 89.97 (81.97; 97.98) | -1.56 (-8.02; 4.89) | 0.6322 | 16 | 78.49 (66.05; 90.94) | | -6.35 (-18.58; 5.87) | 0.3015 |
| Month 24 | 20 | 91.12 (83.03; 99.21) | -0.42 (-6.92; 6.09) | 0.8989 | 4 | 81.22 (60.73; 101.72) | | -3.63 (-23.87; 16.62) | 0.7206 |
| **Mental Health (0-100)** |  |  |  |  |  |  | |  |  |
| Screening | 22 | 80.02 (73.96; 86.08) | **–** | **–** | 17 | 70.05 (62.73; 77.37) | | **–** | **–** |
| Month 6 | 21 | 79.22 (73.11; 85.33) | -0.80 (-6.14; 4.54) | 0.7677 | 19 | 68.00 (60.92; 75.08) | | -2.05 (-9.37; 5.27) | 0.5762 |
| Month 12 | 20 | 80.96 (74.76; 87.16) | 0.94 (-4.45; 6.34) | 0.7294 | 18 | 70.13 (62.92; 77.34) | | 0.08 (-7.38; 7.54) | 0.9826 |
| Month 18 | 21 | 78.87 (72.74; 85.00) | -1.15 (-6.51; 4.21) | 0.6726 | 16 | 69.53 (62.04; 77.02) | | -0.52 (-8.20; 7.16) | 0.8920 |
| Month 24 | 20 | 79.95 (73.74; 86.15) | -0.07 (-5.47; 5.33) | 0.9802 | 4 | 72.38 (59.71; 85.04) | | 2.33 (-10.39; 15.04) | 0.7147 |

EU, European; SF-36v2, Short Form-36 version 2; US, United States.
