# Supplementary figures and images for: Treatment Satisfaction with Subcutaneous Immunoglobulin Replacement Therapy in Patients with Primary Immunodeficiency: a Pooled Analysis of Six Hizentra® Studies
Source: J Clin Immunol. 2018 Nov 21;38(8):886–97. doi: 10.1007/s10875-018-0562-3 (PMC6292975; doi:10.1007/s10875-018-0562-3)

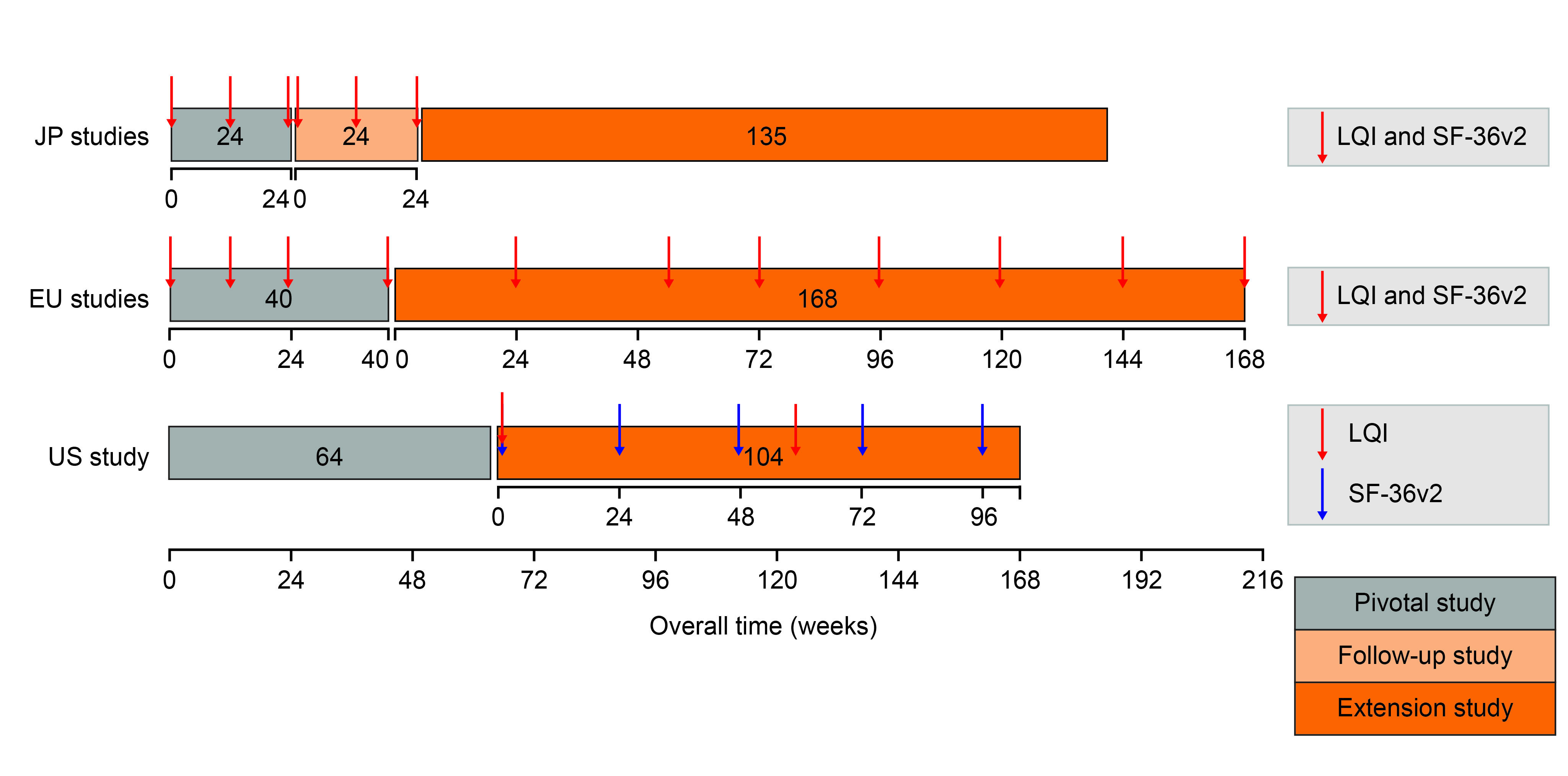

Supplement: Supplementary file 2 — (JPG 1142 kb) [file 10875_2018_562_MOESM2_ESM.jpg]
